# Integration of ultrasound-based radiomics with clinical features for predicting cervical lymph node metastasis in postoperative patients with differentiated thyroid carcinoma

**DOI:** 10.1007/s12020-023-03644-9

**Published:** 2023-12-22

**Authors:** Fengjing Fan, Fei Li, Yixuan Wang, Zhengjun Dai, Yuyang Lin, Lin Liao, Bei Wang, Hongjun Sun

**Affiliations:** 1 Department of Medical Ultrasound, Shandong Provincial Qianfoshan Hospital, Shandong University, Jinan, China; 2 Scientific Research Department, Huiying Medical Technology Co., Ltd, Beijing, China; 3 Department of Endocrinology and Metabology, Shandong Provincial Qianfoshan Hospital, Shandong University, Jinan, China

**Keywords:** Radiomics, Ultrasound, Nomogram, Lymph node metastasis, Differentiated thyroid carcinoma

## Abstract

**Objective:**

The primary objective was to establish a radiomics model utilizing longitudinal +cross-sectional ultrasound (US) images of lymph nodes (LNs) to predict cervical lymph node metastasis (CLNM) following differentiated thyroid carcinoma (DTC) surgery.

**Methods:**

A retrospective collection of 211 LNs from 211 postoperative DTC patients who underwent neck US with suspicious LN fine needle aspiration cytopathology findings at our institution was conducted between June 2021 and April 2023. Conventional US and clinicopathological information of patients were gathered. Based on the pathological results, patients were categorized into CLNM and non-CLNM groups. The database was randomly divided into a training cohort (*n* = 147) and a test cohort (*n* = 64) at a 7:3 ratio. The least absolute shrinkage and selection operator algorithm was applied to screen the most relevant radiomic features from the longitudinal + cross-sectional US images, and a radiomics model was constructed. Univariate and multivariate analyses were used to assess US and clinicopathological significance features. Subsequently, a combined model for predicting CLNM was constructed by integrating radiomics, conventional US, and clinicopathological features and presented as a nomogram.

**Results:**

The area under the curves (AUCs) of the longitudinal + cross-sectional radiomics models were 0.846 and 0.801 in the training and test sets, respectively, outperforming the single longitudinal and cross-sectional models (*p* < 0.05). In the testing cohort, the AUC of the combined model in predicting CLNM was 0.901, surpassing that of the single US model (AUC, 0.731) and radiomics model (AUC, 0.801).

**Conclusions:**

The US-based radiomics model exhibits the potential to accurately predict CLNM following DTC surgery, thereby enhancing diagnostic accuracy.

## Introduction

Differentiated thyroid carcinoma (DTC), mainly including papillary thyroid carcinoma (PTC) and follicular thyroid carcinoma (FTC), accounts for the great majority (>90%) of thyroid cancers and is characterized by indolent growth and low malignancy [1, 2]. The cervical lymph node (LN) is one of the most prone to metastasis after thyroid cancer surgery. Previous studies have shown that approximately 13.3% of patients experience recurrence after primary surgery, which may be related to mortality in a small subset of PTC patients [3–6]. In the postoperative management of thyroid cancer, neck ultrasound (US) plays an important role in thyroid bed recurrence or cervical lymph node metastasis (CLNM).

The US characteristics of CLNM in thyroid cancer have been well demonstrated in the previous literature. These malignant signs include round shape, the absence of echogenic fatty hilum, the presence of cystic area, calcification, hyperechogenicity, abnormal vascularity, and increasing size [7–9]. However, no single US feature is sensitive enough to detect CLNM of thyroid cancer [7]. Moreover, the interpretation of US images is subjective and will be affected by the examiner’s clinical experience. Additionally, changes after thyroidectomy, such as residual thyroid glands, postoperative suture granuloma, fibrosis, reactive hyperplasia of LNs, or fat necrosis, are similar to metastatic cervical LNs on US [10], further increasing the difficulty of diagnosis.

Radiomics has developed rapidly in recent years, with the objectives of extracting many quantitative signatures from medical images, deeply mining tumor biological information, analyzing tumor heterogeneity and facilitating clinical decision-making [11–13]. Radiomics is a quantitative, reproducible, less operator dependent, relatively objective method that compensates for the shortcomings of conventional US. Currently, US-based radiomics is widely used in the thyroid. Previous studies have focused on thyroid nodules or the use of US features of preoperative thyroid cancer primary lesions to predict CLNM [14, 15]. To our knowledge, few studies have explored the US-based radiomic features of LNs to predict CLNM in patients with postoperative DTC.

Therefore, the aim of this study was to investigate the value of US-based radiomics for the prediction of CLNM in postoperative patients with DTC and construct a model combining US-based radiomics and clinical features for predicting CLNM in this population.

## Materials and methods

### Patients

The Ethics Committee of Shandong University Affiliated Qianfoshan Hospital authorized this retrospective study and waived the need to obtain informed consent.

**Fig. 1:**
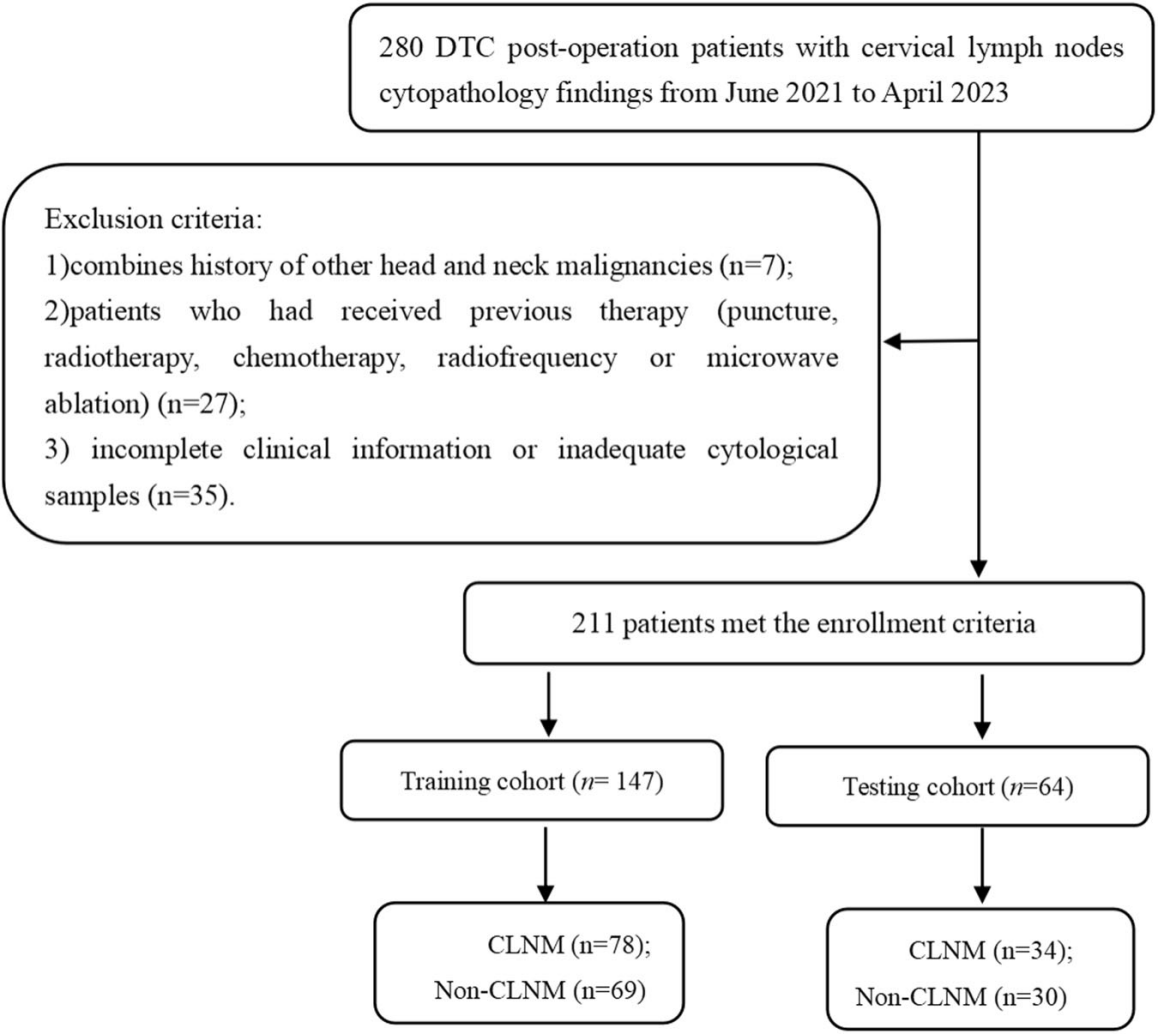
Patient selection flowchart in this study. DTC differentiated thyroid carcinoma, CLNM cervical lymph node metastasis

From June 2021 to April 2023, patients with postoperative DTC who underwent neck US with suspicious LN fine needle aspiration (FNA) cytopathology findings at our institution were retrospectively enrolled. The suspicious LN criteria for FNA in our study were as follows: the absence of a fatty echogenic hilum, alteration of the internal echogenicity (calcification, cystic changes, hyperechogenicity), and a long-to-short-axis ratio ≤2. The selection criteria of patients enrolled were as follows: 1) patients operated on with total thyroidectomy and central region LN dissection, with or without lateral cervical LN dissection; 2) high US image quality, saved in Digital Imaging and Communications in Medicine (DICOM) format; and 3) complete clinical information. The exclusion criteria of patients enrolled were as follows: 1) combined history of other head and neck malignancies; 2) patients who had received previous therapy (puncture, radiotherapy, chemotherapy, radiofrequency or microwave ablation); and 3) incomplete clinical information or inadequate cytological samples. All patients underwent FNA within one week after ultrasonography, and all laboratory findings were obtained before aspiration. If a patient had multiple suspicious LNs, one of the most suspicious LNs was selected for inclusion in the study. A total of 211 LNs from 211 patients (135 females and 76 males, mean age, 45.47 years ±11.81; range, 18–76 years) were enrolled in our study. Figure 1 illustrates a detailed patient screening flowchart.

The clinicopathological features of postoperative DTC patients were collected based on the postoperative pathological results, including the size of the primary thyroid cancer lesion (maximum diameter of the largest node selected for multiple nodes), the presence of Hashimoto’s thyroiditis, extrathyroidal invasion (three grades: no, thyroid capsule invasion and extrathyroidal invasion), multifocality (number of thyroid lesions ≥ 2 defined as multifocal), and the number of lymph node metastasis (LNM) in the central and lateral neck regions (three grades: 0, 1–5, >5). In addition, whether the patient received radioiodine therapy and iodine dose were recorded. All patient laboratory test data were collected, including thyroglobulin (Tg) and antithyroglobulin antibody (TgAb), which were measured by the electrochemiluminescence immunoassay method. With reference to Augusto et al., DTC patients with postoperative suppressed Tg levels ≥1 ng/ml or stimulated Tg levels ≥2 ng/ml or a progressive increase in TgAb on consecutive follow-up examinations were defined as having biochemical recurrence [16].

The total dataset was randomly divided into a training set and a testing set at a ratio of 7:3 using random seed value 52. Table 1 shows the baseline conventional US and clinicopathological information of the training and testing cohort patients.

**Table 1:** Baseline conventional ultrasound and clinicopathological data of patients in the training and test cohorts

Variables	Training cohort (*n* = 147)		*P*-value	Testing cohort (*n* = 64)		*P*-value
	Non-CLNM (*n* = 69)	CLNM (*n* = 78)		Non- CLNM (*n* = 30)	CLNM (*n* = 34)	
Age(years), *n* (%)			0.34			0.69
> 45	40 (58)	38 (49)		13 (43)	12 (35)	
≤45	29 (42)	40 (51)		17 (57)	22 (65)	
Gender, *n* (%)			0.29			0.39
Female	42 (61)	55 (71)		20 (67)	18 (53)	
Male	27 (39)	23 (29)		10 (33)	16 (47)	
Conventional ultrasound features of lymph nodes						
Location, *n* (%)			0.44			0.03
III, IV, VI	54 (78)	66 (85)		20 (67)	31 (91)	
I, II, V, VII	15 (22)	12 (15)		10 (33)	3 (9)	
Long axis, mm, Median (Q1, Q3)	12 (9, 15.8)	11.5 (8.8, 16.0)	0.94	10.8 (9.0, 18.0)	11 (8.8, 14.5)	0.61
Short axis, mm, Median (Q1, Q3)	5.3 (5, 6.6)	6 (5, 8)	0.01	5 (5, 6)	6 (5, 8)	0.004
Long-to-short axis ratio, *n* (%)			0.08			0.16
> 2	31 (45)	23 (29)		14 (47)	9 (26)	
≤2	38 (55)	55 (71)		16 (53)	25 (74)	
Hilus, *n* (%)			0.16			<0.001
Present	9 (13)	4 (5)		9 (30)	0 (0)	
Absent	60 (87)	74 (95)		21 (70)	34 (100)	
Calcification, *n* (%)			<0.001			<0.001
Present	3 (4)	34 (44)		1 (3)	15 (44)	
Absent	66 (96)	44 (56)		29 (97)	19 (56)	
Cystic area, *n* (%)			0.03			0.11
Present	0 (0)	6 (8)		1 (3)	6 (18)	
Absent	69 (100)	72(92)		29 (97)	28 (82)	
Hyperechogenicity, *n* (%)			<0.001			0.08
Present	1 (1)	22 (28)		2 (7)	9 (26)	
Absent	68 (99)	56 (72)		28 (93)	25 (74)	
Abnormal vascularity, *n* (%)			<0.001			0.10
Present	4 (6)	28 (36)		5 (17)	13 (38)	
Absent	65 (94)	50 (64)		25 (83)	21 (62)	
Clinicopathological features of primary thyroid tumors						
Tumor size, mm, Median (Q1, Q3)	12 (8, 22.8)	16 (12.0, 25.0)	0.009	10 (7, 18.5)	16 (11.8, 26.5)	0.02
Extrathyroidal invasion, *n* (%)			<0.001			0.002
No	26 (38)	7 (9)		18 (60)	6 (18)	
Thyroid capsule invasion	29 (42)	40 (51)		9 (30)	20 (59)	
Extrathyroidal invasion	14 (20)	31 (40)		3 (10)	8 (24)	
Multifocality, *n* (%)			0.12			0.251
Positive	41 (59)	57 (73)		19 (63)	27 (79)	
Negative	28 (41)	21 (27)		11 (37)	7 (21)	
Hashimoto’s thyroiditis, *n* (%)			0.52			1.0
Positive	12 (17)	18 (23)		7 (23)	8 (24)	
Negative	57 (83)	60 (77)		23 (77)	26 (76)	
Number of central region LNM, *n* (%)			0.03			0.19
0	15 (22)	6 (8)		5 (17)	4 (12)	
1~5	31 (45)	35 (45)		19 (63)	16 (47)	
> 5	23 (33)	37 (47)		6 (20)	14 (41)	
Number of lateral neck LNM, *n* (%)			0.04			0.07
0	40 (58)	32 (41)		18 (60)	15 (44)	
1~5	19 (28)	22 (28)		10 (33)	9 (27)	
> 5	10 (14)	24 (31)		2 (7)	10 (29)	
Number of courses for 131I therapy, *n* (%)			0.58			0.47
0~1	42 (61)	52 (67)		26 (87)	26 (76)	
≥2	27 (39)	26 (33)		4 (13)	8 (24)	
Cumulative dose of 131I activities, mCi	100 (0, 250)	120 (0, 250)	0.57	0 (0, 100)	50 (0, 185)	0.19
Biochemical recurrence, *n* (%)			<0.001			0.007
No	53 (77)	16 (21)		19 (63)	9 (26)	
Yes	16 (23)	62 (79)		11 (37)	25 (74)	

*CLNM* cervical lymph node metastasis*, LNM* lymph node metastasis

### US image acquisition

US examination was performed using a uniform type of high-resolution US instrument (GE LOGIQ E9) with a linear array probe (6–15 MHz). Then, a specific “thyroid” procedure was used to examine the thyroid bed and bilateral cervical LNs, and the location, size, internal echogenicity, presence of hilus, calcification, cystic changes and hyperechogenicity of the suspected LNs were recorded. Color Doppler flow imaging was used to observe the LN vascular distribution.

The locations of the suspected LNs were determined based on the guidelines from the American Head and Neck Society and the American Academy of Otolaryngology-Head and Neck Surgery [17]. LN size was measured as the long-axis and short-axis diameter on the maximal longitudinal section. Calcifications included microcalcifications and macrocalcifications. The presence of echo-free areas in the LNs was defined as cystic changes. Hyperechogenicity was defined as the cortex echogenicity of LNs being higher than that of adjacent muscles. Abnormal vascularity was the presence of abundant, peripheral or mixed blood flow. The conventional US images of LNs were evaluated independently by two sonographers with at least 5 years of diagnostic experience who were blind to the pathological results. If there was any disagreement, the two radiologists would make a decision by consensus.

### US image segmentation and feature extraction

Anonymized US grayscale DICOM images of LNs, captured in both longitudinal and cross-sectional images, were securely uploaded to the RadCloud platform (version 7.2; Huiying Medical Technology Co., Ltd, Beijing, China, https://mics.huiyihuiying.com). Two experienced radiologists, with over 5 years of professional experience, conducted the image analysis without access to the pathological outcomes, ensuring unbiased evaluation. The radiologists manually performed region of interest (ROI) segmentation, meticulously outlining the LNs. The ROIs consisted of two distinct components: longitudinal and cross-sectional segments. Subsequently, relevant feature values were extracted from the segmented ROIs. To ensure the precision and dependability of the results, an experienced radiologist (with 8 years of work experience) conducted thorough validation and correction of all delineated labels.

Radiomics feature extraction was then performed on this platform. Specifically, the platform utilized the PyRadiomics package (version 3.1.0, https://pyradiomics.readthedocs.io/), based on the Python language (version 3.7.0, https://www.python.org), to extract a comprehensive set of radiomics features from medical US images. To ensure standardization, preprocessing steps of the US images, including image resampling and grayscale discretization, were performed. A total of 661 image features were extracted from each ROI, encompassing seven major categories. These categories included first-order statistics, 2D shape features, gray-level cooccurrence matrix (GLCM) features, gray-level run-length matrix (GLRL) features, gray-level size zone matrix (GLSZM) features, gray-level dependence matrix (GLDM) features, and neighboring gray-tone difference matrix (NGTDM) features.

### Feature selection

Prior to selecting radiomics features, *Z*-score normalization was performed on all features. To mitigate the risk of overfitting and enhance the relevance of features, dimensionality reduction techniques were employed. Initially, the intraclass correlation coefficient (ICC) was utilized to evaluate the interobserver reproducibility of the radiomics features. Features with ICC values > 0.8 were considered reliable and were included in the subsequent feature selection process. Subsequently, a variance threshold of 0.8 and the Select K Best method based on univariate analysis were employed. Last, the least absolute shrinkage and selection operator (LASSO) regression method was used for feature selection utilizing 10-fold cross-validation.

### Development and validation of the model

Radiomics models were developed using longitudinal section, cross-section, and combined longitudinal + cross-sectional images of LNs to distinguish cervical LN status. Logistic regression (LR), random forest (RF), support vector machine (SVM), and decision tree (DT) algorithms were employed to create radiomics models, and then the best performing model was determined.

Significant US features from the training cohort were incorporated into multivariate analysis to construct US models in the training and test cohorts. Furthermore, considering the potential influences of clinicopathological characteristics, we performed multivariate analysis to integrate radiomic scores (Rad-scores), conventional US features, and independent clinical risk factors. Subsequently, a nomogram for predicting CLNM was constructed based on the developed model to facilitate clinical decision-making.

**Fig. 2:**
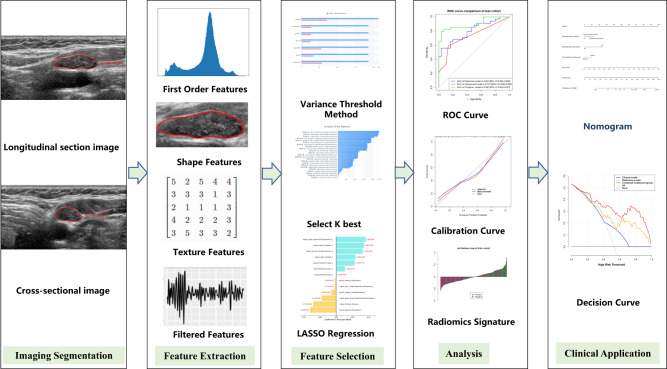
The workflow diagram of this study. ROC receiver operating characteristic, LASSO the least absolute shrinkage and selection operator

The Hosmer‒Lemeshow goodness-of-fit test and calibration curve were constructed to assess the performance of the prediction model. Figure 2 illustrates the workflow diagram depicting the various stages of this study.

### Statistical analysis

Student’s *t* test and Mann‒Whitney U test were utilized to compare the differences between groups in measurement data. Count data comparisons between groups were made using the Chi-square test or Fisher’s exact probability method. Univariate and multivariate logistic regression analyses were utilized to screen US and clinicopathological characteristics. The area under the curve (AUC) estimates of different prediction models were compared using the nonparametric DeLong’s test. SPSS 20 (IBM Corp., Armonk, NY) and R software (version 4.2.1; https://www.r-project.org/) were used for statistical analysis. A two-sided probability value (*p* value) < 0.05 was considered statistically significant.

## Results

### Clinical and ultrasound characteristics

The 211 patients included 208 with PTC and 3 with FTC. Among PTCs, there were 197 low-risk subtypes (190 classical variants, 7 follicular variants) and 11 high-risk subtypes (5 diffuse sclerosing variants, 4 tall cell and 2 columnar cell subtypes), and no significant differences were found between low-risk and high-risk subtypes in predicting CLNM. Table 2 presents the results of univariate and multivariate analyses predicting CLNM in the training cohort. In terms of US features, compared with the non-CLNM group, the CLNM group mostly showed calcification (*p* < 0.001), cystic area (*p* = 0.02), hyperechogenicity (*p* < 0.001) and abnormal vascularity (*p* < 0.001). In addition, the short axis of LNs in the CLNM group was significantly higher than that in the non-CLNM group (*p* = 0.02).

**Table 2:** Results of the univariate and multivariate analysis based on the training cohort

Variables	Univariate analysis			Multivariate analysis	
	Non-CLNM (*n* = 69)	CLNM (*n* = 78)	*P*-value	Odds ratio (95%CI)	*P*-value
Age(years), *n* (%)			0.26		
> 45	40 (58)	38 (49)			
≤45	29 (42)	40 (51)			
Gender, *n* (%)			0.22		
Female	42 (61)	55 (71)			
Male	27 (39)	23 (29)			
Conventional ultrasound features of lymph nodes					
Location, *n* (%)			0.32		
III, IV, VI	54 (78)	66 (85)			
I, II, V, VII	15 (22)	12 (15)			
Long axis, mm, Median (Q1, Q3)	12 (9, 15.8)	11.5 (8.8, 16.0)	0.69		
Short axis, mm, Median (Q1, Q3)	5.3 (5, 6.6)	6 (5, 8)	0.02*	1.08 (0.88,1.32)	0.45
Long-to-short axis ratio, *n* (%)			0.05		
> 2	31 (45)	23 (29)			
≤2	38 (55)	55 (71)			
Hilus, *n* (%)			0.09		
Present	9 (13)	4 (5)			
Absent	60 (87)	74 (95)			
Calcification, *n* (%)			<0.001*	9.12 (2.46, 33.75)	0.001**
Present	3 (4)	34 (44)			
Absent	66 (96)	44 (56)			
Cystic area, *n* (%)			0.02*	NA	0.99
Present	0 (0)	6 (8)			
Absent	69 (100)	72(92)			
Hyperechogenicity, *n* (%)			<0.001*	10.78 (1.26, 92.37)	0.03**
Present	1 (1)	22 (28)			
Absent	68 (99)	56 (72)			
Abnormal vascularity, *n* (%)			<0.001*	2.81 (0.76, 10.39)	0.12
Present	4 (6)	28 (36)			
Absent	65 (94)	50 (64)			
Clinicopathological features of primary thyroid tumors					
Tumor size, mm, Median (Q1, Q3)	12 (8, 22.75)	16 (12.0, 25.0)	0.03*	1.00 (0.96, 1.04)	0.95
Extrathyroidal invasion, *n* (%)					
No	26 (38)	7 (9)			
Thyroid capsule invasion	29 (42)	40 (51)	0.001*	6.13 (1.85, 20.31)	0.003**
Extrathyroidal invasion	14 (20)	31 (40)	<0.001*	6.28 (1.71, 23.10)	0.006**
Multifocality, *n* (%)			0.08		
Positive	41 (59)	57 (73)			
Negative	28 (41)	21 (27)			
Hashimoto’s thyroiditis, *n* (%)			0.40		
Positive	12 (17)	18 (23)			
Negative	57 (83)	60 (77)			
Number of central region LNM, *n* (%)					
0	15 (22)	6 (8)			
1~5	31 (45)	35 (45)	0.06	1.46 (0.40, 5.31)	0.57
> 5	23 (33)	37 (47)	0.01*	1.75 (0.43, 7.08)	0.43
Number of lateral neck LNM, *n* (%)					
0	40 (58)	32 (41)			
1~5	19 (28)	22 (28)	0.35	0.71 (0.26, 1.97)	0.51
> 5	10 (14)	24 (31)	0.01*	0.83 (0.24, 2.85)	0.77
Number of courses for 131I therapy, *n* (%)			0.47		
0~1	42 (61)	52 (67)			
≥2	27 (39)	26 (33)			
Cumulative dose of 131I activities, mCi	100 (0, 250)	120 (0, 250)	0.92		
Biochemical recurrence, *n* (%)			<0.001*	13.64 (5.44, 34.21)	<0.001**
No	53 (77)	16 (21)			
Yes	16 (23)	62 (79)			

*CLNM* cervical lymph node metastasis*, LNM* lymph node metastasis, *CI* confidence interval, *NA* values were not available

*P-value of univariate logistic regression analysis <0.05

**P-value of multivariate logistic regression analysis <0.05

Based on postoperative pathological features of the thyroid primary lesion, the incidence of extrathyroidal invasion (*p* ≤ 0.001) and the number of LNMs >5 in the central and lateral neck regions were substantially higher in the CLNM group (*p* = 0.01) than in the non-CLNM group. Moreover, the primary tumor size in the CLNM group was significantly larger than that in the non-CLNM group (*p* = 0.03). Additionally, patients with biochemical recurrence were more likely to present with CLNM (*p* < 0.001). Among 114 patients with biochemical recurrence, 83 had elevated Tg only, 26 had elevated TgAb only, and 5 exhibited elevations of both Tg and TgAb.

Multivariate analysis revealed that US features (calcification, hyperechogenicity) and clinicopathological features (extrathyroidal invasion, biochemical recurrence) were independent factors for predicting CLNM.

### Radiomics results and model building

**Fig. 3:**
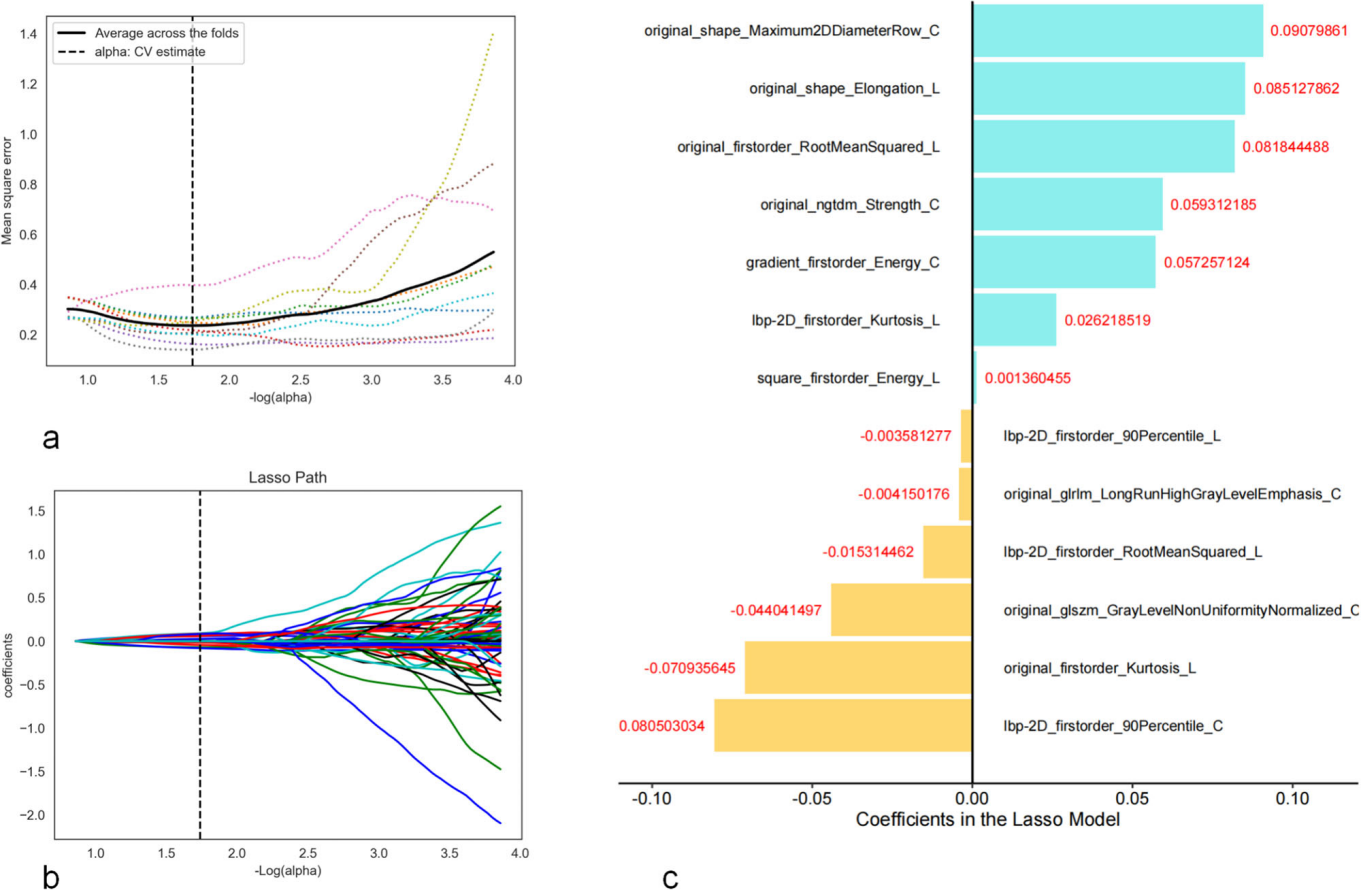
This study uses the least absolute shrinkage and selection operator (LASSO) in the parametric method to select the ultrasound image radiomics features. **a** Select the optimal alpha of 0.0183 with log(alpha) of −1.738; **b** LASSO coefficient profiles of the 224 selected features. **c** A Rad-score was built using 13 screened features and their corresponding coefficients

In our study, a total of 1322 feature values were extracted from the longitudinal + cross-sectional images of LNs. After data dimensionality reduction, 13 nonzero coefficient radiomics features were obtained (6 from cross-sections and 7 from longitudinal sections). These features were applied to compute the total cohort per patient Rad-score, which was formulated by calculating the sum of the corresponding feature values multiplied by their respective weights as illustrated in Fig. 3.

A combined radiomics feature machine learning model was constructed with LR, SVM, RF and DT, and the best overall performance of the LR model was shown (Table 3). The AUCs of the LR model were 0.846 and 0.801 in the training and test cohorts, respectively, and the accuracy, sensitivity and specificity of Rad-scores were 0.762, 0.731, 0.797 and 0.688, 0.676, 0.700 in the training and test sets, respectively, showing that radiomics had promising predictive value for CLNM.

**Table 3:** The performance of LR, SVM, DT and RF in longitudinal + cross-sectional radiomics for predicting lymph node status after differentiated thyroid carcinoma

Cohort	ML model	AUC	Accuracy	Sensitivity	Specificity
Train	LR	0.846	0.762	0.731	0.797
	SVM	0.868	0.796	0.795	0.797
	DT	0.821	0.741	0.590	0.913
	RF	0.903	0.844	0.782	0.913
Test	LR	0.801	0.688	0.676	0.700
	SVM	0.734	0.672	0.735	0.600
	DT	0.734	0.625	0.382	0.900
	RF	0.674	0.609	0.529	0.700

*ML* machine learning*, LR* logistic regression, *SVM* support vector machine, *DT* decision tree, *RF* random forest, *AUC* area under the curve

Based on the methodology mentioned above, 8 radiomics features were extracted from 661 longitudinal section imaging signatures to calculate the Rad-score of longitudinal sections, and 8 radiomics features were extracted from 661 cross-sectional imaging signatures to calculate the Rad-score of cross-sectional sections. Notably, the features screened from longitudinal sections and cross-sections alone were different from those screened from the combination of longitudinal +cross-sectional sections. LR was used to construct the radiomics model, and the results showed that the longitudinal +cross-sectional radiomics models outperformed the single longitudinal section and cross-section models (Table 4).

**Table 4:** The performance of lymph nodes longitudinal, cross-sectional and longitudinal + cross-sectional radiomics model using logistic regression for predicting lymph node status after differentiated thyroid carcinoma

Cohort	Model	AUC	Accuracy	Sensitivity	Specificity
Train	Longitudinal section	0.782	0.673	0.628	0.724
	Cross-section	0.760	0.701	0.654	0.754
	Longitudinal +cross-section	0.846	0.762	0.731	0.797
Test	Longitudinal section	0.768	0.703	0.676	0.733
	Cross-section	0.698	0.641	0.647	0.633
	Longitudinal +cross-section	0.801	0.688	0.676	0.700

### Construction and validation of the model

**Fig. 4:**
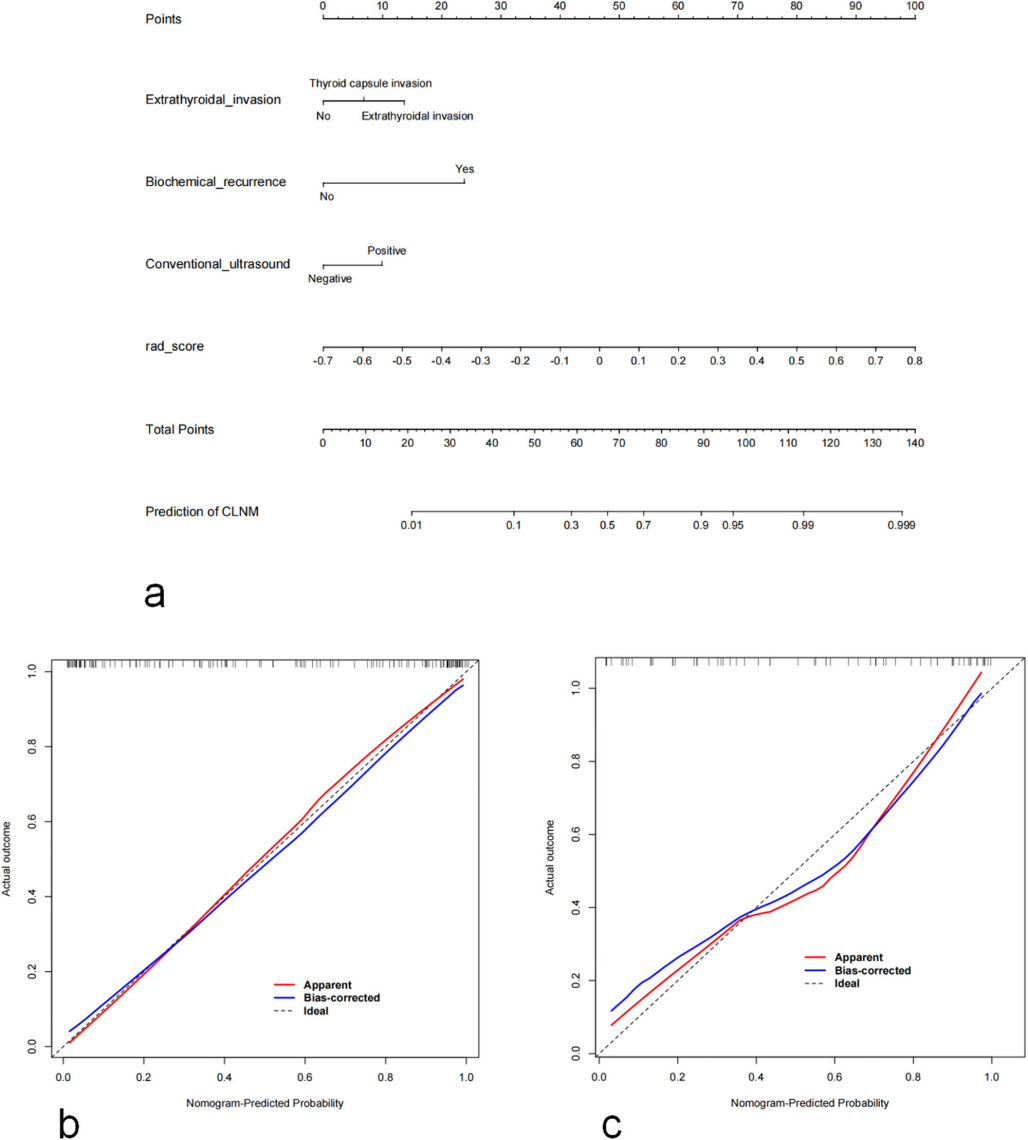
A nomogram combining Rad-score, conventional ultrasound, extrathyroidal invasion and biochemical recurrence was developed to predict the risk of CLNM in patients with postoperative differentiated thyroid cancer (**a**). Nomogram calibration curves for the training (**b**) and test cohorts (**c**)

In our research, a model combining conventional US, Rad-scores and clinical features was established and presented as a nomogram (Fig. 4). Then, to assess the model performance, the Hosmer–Lemeshow goodness of fit test was applied to the training and test cohorts, with *p* values of 0.18 and 0.25, respectively, indicating that no significant differences were found between the model-predicted risk of CLNM and the actually occurring risk. The calibration curve of the radiomics nomogram showed good agreement between the predicted and actual CLNM in the training and test sets (Fig. 4).

**Fig. 5:**
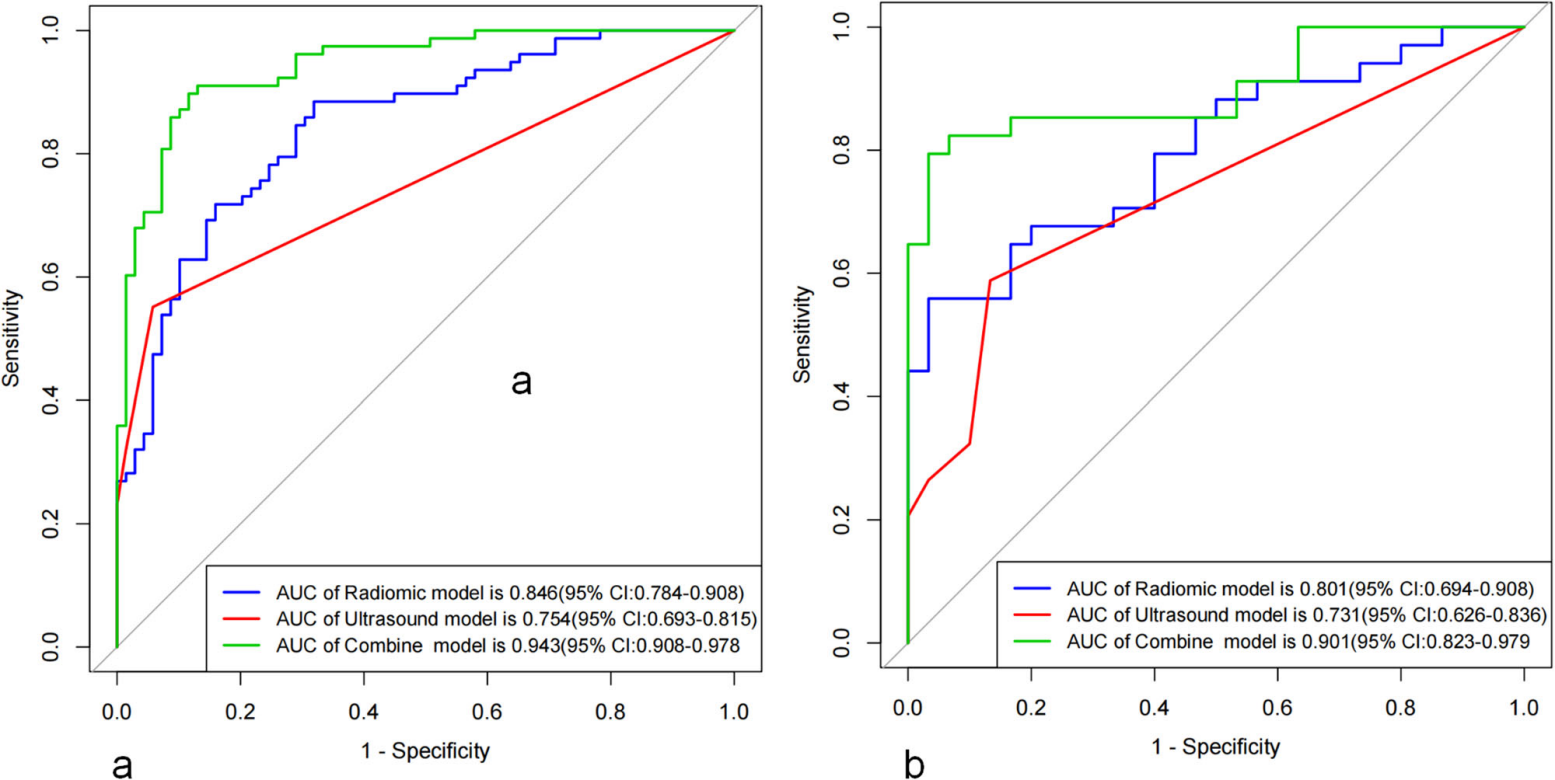
Receiver operating characteristic (ROC) curves of the combine model, conventional ultrasound model, and radiomics model were compared in the training (**a**) and test (**b**) cohorts to predict the risk of CLNM in patients with postoperative differentiated thyroid cancer

Compared with conventional US and radiomics models, the combined model showed the best performance in predicting CLNM. Table 5 shows the performance evaluation of each model. As Fig. 5 shows, the receiver operating characteristic (ROC) curves corresponding to the three models and the AUCs of the combined model in the training and test cohorts were 0.943 and 0.901, respectively. DeLong’s test indicated that the radiomics model was significantly better than the conventional US model (*p* < 0.05), and the combined model was significantly better than the single US model and radiomics model (*p* < 0.05).

**Table 5:** The performance of conventional ultrasound, longitudinal +cross-sectional, and combined model for predicting lymph node status after differentiated thyroid carcinoma

Cohort	Model	AUC	Accuracy	Sensitivity	Specificity
Train	Conventional ultrasound	0.754	0.735	0.551	0.942
	Longitudinal +cross-section	0.846	0.762	0.731	0.797
	Combined model	0.943	0.878	0.872	0.884
Test	Conventional ultrasound	0.731	0.719	0.588	0.867
	Longitudinal +cross-section	0.801	0.688	0.676	0.700
	Combined model	0.901	0.844	0.824	0.867

**Fig. 6:**
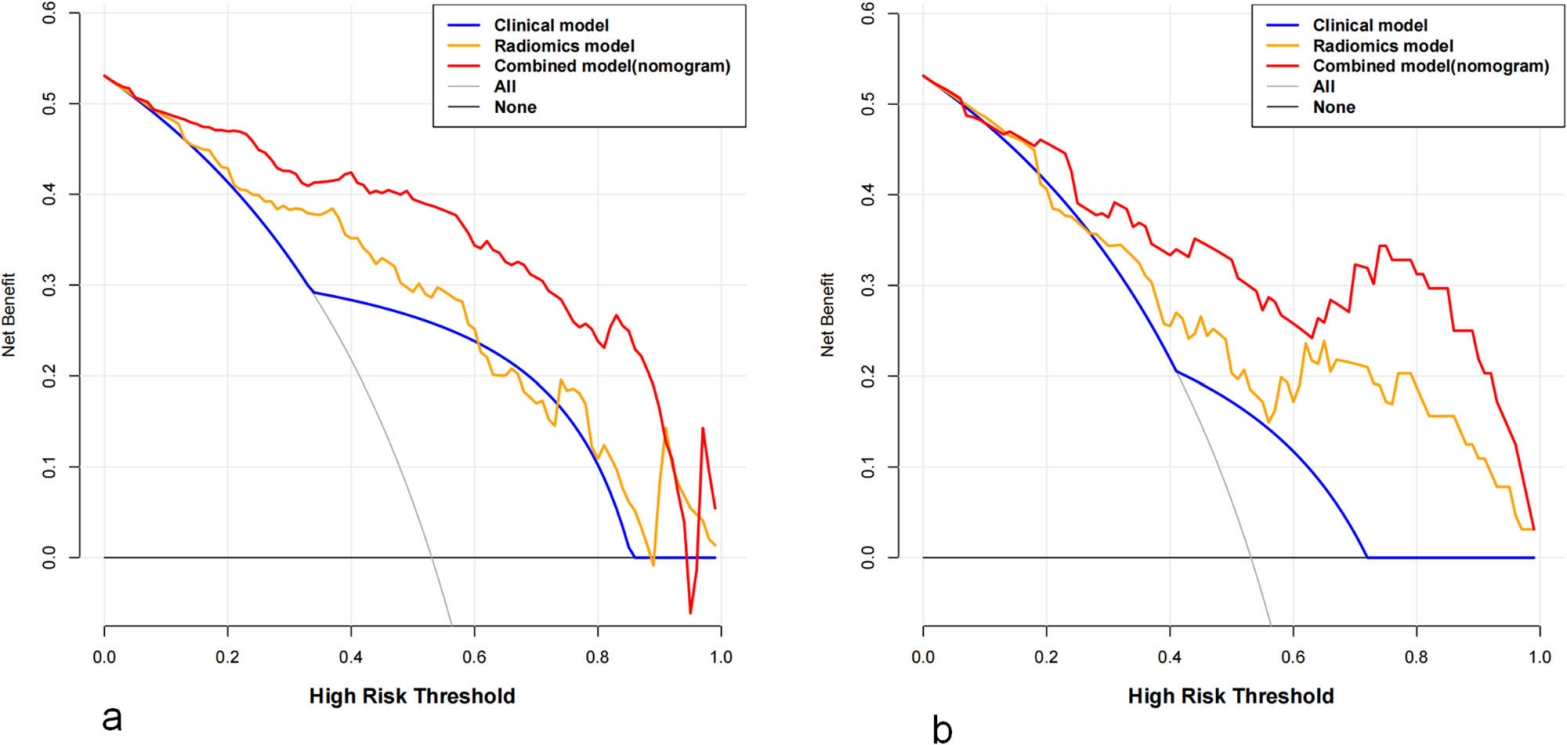
Decision curve analysis was conducted for each model in the training (**a**) and test cohorts (**b**)

The decision curve analysis suggested that the combined model yielded the highest net benefit in predicting CLNM compared to the radiomic and US models (Fig. 6). In patients with postoperative DTC, the combination of US-based radiomics with clinical features showed greater efficacy in predicting the status of the cervical LNs.

## Discussion

The most common pathological subtype of DTC is PTC, and lymphatic metastasis is its main metastasis mode. The most common site of recurrence after DTC surgery is also cervical LNs, and high-frequency US has become an important tool to evaluate postoperative locoregional recurrence. This study showed that US characteristics of LNs, including short axis, calcification, cystic area, hyperechogenicity and abnormal vascularity, were risk factors for predicting CLNM, similar to previous study findings [18, 19]. No significant differences were found in terms of LN hilus (*p* = 0.09), LN long axis (*p* = 0.69) or long-to-short axis ratio (*p* = 0.05) between the non-CLNM and CLNM groups. This was because only suspicious LNs were included in this study, and LNs with normal LN US features were not included. Moreover, including data from such LNs could also reduce the overall diagnostic accuracy.

Recently, previous studies have shown that radiomics features enable objective and quantitative analysis that can provide insights for personalized medicine and potentially improve the accuracy of diagnosis, prognosis, and prediction [20, 21]. US-based radiomics studies have also shown critical value in predicting the prognosis of many diseases, such as breast, thyroid and liver cancer [15, 22–24]. Previous studies have mostly focused on preoperatively predicting CLNM status using the US radiomics features of primary thyroid tumors. Vivian et al.‘s study showed the potential of using the US radiomics signature of the primary tumor alone to predict lateral neck region LNM in PTC patients, with AUCs of 0.710 and 0.62 in the training and test sets, respectively [25].

This study innovatively used US radiomics of cervical LNs to determine LN status after DTC surgery. We preliminarily found that the radiomics of both longitudinal and cross-sectional images of cervical LNs had good diagnostic performance in predicting CLNM, and the diagnostic performance of the longitudinal + cross-sectional radiomics model was superior. The AUCs of the radiomics model in predicting CLNM were 0.846 and 0.801 in the training and test cohorts, respectively, which were better than those of conventional US, suggesting that US-based radiomics analysis had good application value in predicting CLNM after DTC.

Lin et al. showed that grayscale US radiomics analysis of LNs had potential value in extracting significant US features and enhancing the diagnosis of CLNM in patients with nasopharyngeal carcinoma, with an AUC of 0.810 (0.739–0.881) in the radiomics model [26]. Additionally, in some studies, US techniques such as elastography, contrast-enhanced US and radiomics have been used to predict the risk of CLNM of PTC preoperatively. The clinical-radiomics nomogram constructed by Li et al. based on contrast-enhanced US for the preoperative prediction of LNM in PTC showed good performance in the training set (AUC = 0.820) and test set (AUC = 0.814) [14]. The study by Meng et al. showed that a radiomics nomogram based on shear-wave elastography had greater discriminatory efficacy in the US-reported LN-negative subgroup (AUC = 0.812) [15].

This study showed that extrathyroidal invasion of the primary thyroid tumor was independently related to CLNM, similar to previous studies [27]. In addition, postoperative serum Tg and TgAb levels are of great value in monitoring recurrence after total thyroidectomy for DTC [7, 28]. Our study findings showed that the biochemical recurrence rate was significantly higher in the CLNM group than in the non-CLNM group. Consequently, in cases where biochemical recurrence is clinically indicated, the sonographer should conduct a thorough neck examination to carefully assess CLNM.

Accounting for the influence of clinical factors in each patient, our study integrated conventional US, Rad-scores, extrathyroidal invasion, and biochemical recurrence into a comprehensive combined model for predicting CLNM. This combined model was then represented as a nomogram to aid clinical decision-making. Remarkably, when compared with the US and radiomics models, the combined model exhibited superior diagnostic performance, achieving AUCs of 0.943 and 0.901 in the training and test sets, respectively. These results underscore the potential clinical utility of our combined model in accurately predicting CLNM in postoperative patients with DTC.

Nonetheless, several limitations need to be recognized in our study. First, since this was a single-center retrospective study with a relatively small sample size, it is essential to conduct prospective multicenter investigations with larger cohorts to validate and strengthen the findings. Second, our analysis focused solely on static grayscale US images of LNs, and the integration of multimodal images, such as radiomics combined with elastography or contrast-enhanced US, could offer additional information and provide superior performance. Therefore, future research will include the acquisition of multimodal images for comprehensive analysis. Third, due to the limited sample size, the classification of LN size was not conducted in detail, warranting further exploration in future studies. Addressing these limitations will be critical for advancing the understanding and clinical application of our findings.

In conclusion, our preliminary study indicates that the identified radiomic features have the potential to serve as predictors of cervical LN status after DTC surgery. The integration of US-based radiomics with clinical features demonstrated enhanced diagnostic efficacy and significantly improved discriminatory performance in evaluating CLNM among patients with postoperative DTC.

## Abbreviations

DTC: differentiated thyroid carcinoma
PTC: papillary thyroid carcinoma
FTC: follicular thyroid carcinoma
LN: lymph node
CLNM: cervical lymph node metastasis
FNA: fine needle aspiration
DICOM: digital Imaging and Communications in Medicine
LNM: lymph node metastasis
Tg: thyroglobulin
TgAb: antithyroglobulin antibody
ROI: region of interest
ICC: intraclass correlation coefficient
LASSO: least absolute shrinkage and selection operator
LR: logistic regression
RF: random forest
SVM: support vector machine
DT: decision tree
AUC: area under the curve
ROC: receiver operating characteristic
